# Safety and Immunogenicity of Betuvax-CoV-2, an RBD-Fc-Based SARS-CoV-2 Recombinant Vaccine: Preliminary Results of the First-in-Human, Randomized, Double-Blind, Placebo-Controlled Phase I/II Clinical Trial

**DOI:** 10.3390/vaccines11020326

**Published:** 2023-02-01

**Authors:** Aleksandr V. Kudriavtsev, Anna V. Vakhrusheva, Nickolay A. Kryuchkov, Maria E. Frolova, Konstantin A. Blagodatskikh, Taras V. Ivanishin, Milana Djonovic, Ekaterina A. Romanovskaya-Romanko, Anton N. Kovalenko, Dmitry A. Lioznov, Tatiana G. Zubkova, Svetlana V. Teplykh, Rodion A. Oseshnyuk, Marina A. Stukova, Artur A. Isaev, Igor V. Krasilnikov

**Affiliations:** 1 Betuvax LLC, 121096 Moscow, Russia; 2 Clinical Excellence Group LLC, 127051 Moscow, Russia; 3 PJSC Human Stem Cells Institute, 129110 Moscow, Russia; 4 Center of Genetics and Reproductive Medicine “Genetico”, 119333 Moscow, Russia; 5 Department of Vaccinology, Smorodintsev Research Institute of Influenza, Ministry of Health of the Russian Federation, 197376 Saint Petersburg, Russia; 6 Professor Clinic LLC, 614070 Perm, Russia; 7 Ecosafety Center LLC, 195000 Saint Petersburg, Russia; 8 Biotechnology Developments LLC, 119285 Moscow, Russia

**Keywords:** COVID-19, SARS-CoV-2, subunit vaccine, betulin, clinical trial, Betuvax-CoV-2

## Abstract

COVID-19, being a life-threatening infection that evolves rapidly, remains a major public health concern calling for the development of vaccines with broad protection against different pathogenic strains and high immunogenicity. Aside from this, other concerns in mass immunization settings are also the scalability of production and relative affordability of the technology. In that regard, adjuvanted protein vaccines with particles mimicking the virus stand out among known vaccine technologies. The “Betuvax-CoV-2” vaccine, developed on the basis of a recombinant protein and an adjuvant, has already been tested in preclinical studies and has advanced to clinical evaluation. Open, double-blinded, placebo-controlled, randomized phase I/II clinical trial of the “Betuvax-CoV-2,” recombinant protein subunit vaccine based on the S-protein RBD fused with the Fc-fragment of IgG, was conducted to evaluate safety and immunogenicity in response to the vaccination. Methods: In the phase I/II clinical trial, 116 healthy adult men and women, ages 18–58, were enrolled: 20 in Stage I, and 96 in Stage II. In Stage I, 20 µg of the vaccine was administered intramuscularly on day 2, and either 5 µg (group 1) or 20 µg (group 2) on day 30. In Stage II, 20 µg of the vaccine was administered intramuscularly on day 2, and either 5 µg (group 3) or 20 µg (group 4) on day 30. In group 5, both injections were replaced with placebo. The primary outcome measures were safety (number of participants with adverse events throughout the study) and antigen-specific humoral immunity (SARS-CoV-2-specific antibodies measured by ELISA and CMIA). Antigen-specific cell-mediated immunity and changes in neutralizing antibodies (detected with a SARS-CoV-2 neutralization assay) were measured as a secondary outcome. The trial is registered with ClinicalTrials.gov (Study Identifier: NCT05270954). Findings: Both vaccine formulations (20 µg + 5 µg and 20 µg + 20 µg) were safe and well tolerated. Most adverse events were mild, and no serious adverse events were detected. On day 51,anti-SARS-CoV-2 total and IgG antibody titers and anti-SARS-CoV-2 neutralizing antibodies were significantly higher in the vaccine groups (both formulations) than in the placebo. A more pronounced CD4+-mediated immune response was observed in the group of volunteers administered with the 20 + 20 μg vaccine formulation. Interpretations: RBD-Fc-based COVID-19 “Betuvax-CoV-2” vaccine in doses (20 + 5 µg and 20 + 20 µg) demonstrated an excellent safety profile and induced a strong humoral response. Further research on the protective effectiveness of the “Betuvax-CoV-2” vaccine for the prevention of COVID-19 is on its way.

## 1. Introduction

COVID-19 remains a life-threatening infection, even after two years since its emergence. Out of six million people worldwide who have died from COVID-19, approximately one million died in the first half of 2022 [1]. A high viral transmission rate could lead to a quick virus evolution which may result in a strain with a higher transmissibility and/or virulence [2]. That said, novel mutant strains of the SARS-CoV-2, such as Omicron, can reinfect even those individuals who previously established immunity against the virus through disease or vaccination. Therefore, it is critical to maintain a high titer of SARS-CoV-2-neutralizing antibodies through revaccination.

The “Betuvax-CoV-2” vaccine was developed as a subunit vaccine containing a recombinant RBD-Fc fusion protein absorbed on the surface of the betulin-based spherical nanoparticles (“Betuspheres”) mimicking the virus and enhancing the immune response [3]. Preclinical studies [4] showed a high safety profile of the “Betuvax-CoV-2” vaccine in various animal models, high titers of neutralizing antibodies, and protectivity against the virus infection and lung damage. In this study, we aim to assess the safety and immunogenicity of the novel subunit COVID-19 vaccine “Betuvax-CoV-2” in Russian adults ages 18−58.

## 2. Methods

### 2.1. Study Design and Participants

A double-blinded, placebo-controlled, randomized phase I/II study was conducted in three investigational centers: the Department of Vaccinology at The Smorodintsev Research Institute of Influenza of the Ministry of Health of the Russian Federation (Saint Petersburg, Russia); the “Eco-Safety” R&D center (Saint Petersburg, Russia); the Center of Professional Medicine (Perm, Russia). The study included 116 healthy male and female volunteers, ages 18–58, who met the inclusion/exclusion criteria with no previous history of COVID-19 or COVID-19 vaccination (Table S1).

This article presents the results of interim data analysis. Statistical analysis on the safety and tolerability in Stages I and II, and those on immunogenicity and efficacy in Stage II was performed for Visits 0–18 (for the period until 3 weeks after the 2nd dose of the vaccine/placebo). Data analysis on the immunogenicity and efficacy in Stage I was performed for Visits 0–19 (for the period until 90 ± 5 days after the 1st dose of the vaccine/placebo).

The clinical trial was approved by the Ethics Council of the Ministry of Health of Russia (Resolution #287 of 14 September 2021), the Ministry of Health of Russia (Approval #581 of 27 September 2021), and the Independent Ethics Committees of three study sites. The study protocol was registered with ClinicalTrials.gov (Study Identifier: NCT05270954).

### 2.2. Randomization and Masking

In Stage I, the volunteers who passed the screening were assigned to Group 1 (G1) and 2 (G2) (*n* = 10 each). In Stage II, healthy volunteers were randomized into Group 3 (G3), 4 (G4), and 5 (G5) (*n* = 32 each). All participants provided their written informed consent before the enrollment.

The volunteers who met the inclusion criteria and did not meet the exclusion criteria were assigned a three-digit Randomization Sequence Number (RSN). The Study Stage I was open-label, while Stage II was double-blind.

### 2.3. Formulations and Dosages of the Test Drug

Betuvax-CoV-2 vaccine suspension for intramuscular injection as 10 μg/mL, 0.5 mL (1 dose): recombinant receptor-binding protein of the SARS-CoV-2 virus (RBD-Fc) as an active ingredient (5 μg); corpuscular adjuvant based on natural betulin (200 μg) and tris(hydroxymethyl)aminomethane (3 mg) as excipients, sodium chloride (4.4 mg), water for injection (up to 0.5 mL).

Betuvax-CoV-2 suspension for intramuscular injection as 40 μg/mL, 0.5 mL (1 dose): recombinant receptor-binding protein of the SARS-CoV-2 virus (RBD-Fc) as an active ingredient (20 μg); corpuscular adjuvant based on natural betulin (200 μg) and tris(hydroxymethyl)aminomethane (3 mg) as excipients, sodium chloride (4.4 mg), water for injection (up to 0.5 mL).

### 2.4. Procedures

The vaccination study schedule included two intramuscular injections (0.5 mL each) within a 28-day interval.

Stage I: Group 1 (*n* = 10) received the vaccine at 20 μg of the active ingredient (first dose) and 5 μg (second dose). Group 2 (*n* = 10) received the vaccine at 20 μg (first dose) and 20 μg (second dose).

Stage II: Group 3 (*n* = 32) received the vaccine at 20 μg of the active ingredient (first dose) and 5 μg (second dose). Group 4 (*n* = 32) received the vaccine at 20 μg (first dose) and 20 μg (second dose). Group 5 (*n* = 32) received two 0.5 mL intramuscular placebo injections of 0.9% sodium chloride solution.

Participation of each volunteer in the study assumed Visit 0 (screening), Visits 1–4 and Visits 10–13 in an inpatient setting (in hospital), and Visits 5–9, 14–20 in an outpatient setting. Volunteers received the study drug (one of two doses) or placebo on Visits 2 and 11.

The study participants were monitored for the expected outcomes. The main safety and tolerability outcomes were centrally reviewed by the Independent Data Monitoring Committee (IDMC).

Total immunoglobulins were determined using the ELISA “SARS-CoV-2-CoronaPass test system” (Biopalitra, Moscow, Russia). The titer of total IgG was measured with chemiluminescent microparticle immunoassay (CMIA) “SARS-CoV-2 IgG II Quant assay” (Abbott Laboratories, Chicago, IL, USA). Neutralizing antibodies were measured by “SARS-CoV-2 Surrogate Virus Neutralization Test Kit” (GenScript, Piscataway, NJ, USA).

### 2.5. Cell Immunity Assessment

Flow cytometry: cryopreserved PBMCs (Peripheral Blood Mononuclear Cells) were thawed in a water bath (+37 °C), washed with 10% FBS (Gibco) and 1% penicillin/streptomycin RPMI medium (Biolot), and seeded into 96-well plates at 1 × 10^6^ cells/100 µL. To stimulate an antigen-specific T-cell response, a mixture of overlapping peptides (15 aa) covering the S-protein sequence of the SARS-CoV-2 virus (PepTivator^®^ SARS-CoV-2 Prot S, Miltenyi Biotec) was used. Antibodies to CD28/CD49b (BD Bioscience) were used as costimulators. For non-specific stimulation of cytokine production (positive stimulation control), a mixture of PMA and Iomomycin (Sigma) was used at a concentration of 50 ng/mL and 1 μg/mL, respectively. To block cell transport, Brefeldin A (BD Bioscience) was used, which was added to the medium simultaneously with specific and nonspecific inducers 16 h before staining. The negative stimulation control included all of the listed components, with the exception of specific (inactivated influenza viruses) and non-specific (PMA/Iomomycin) inducers of the immune response. Staining of surface and intracellular antigens was performed using the Cytofix/Cytoperm reagent kit (BD Bioscience) according to the manufacturer’s instructions. Data collection was performed on a Cytoflex flow cytometer (Beckman Coulter). Gating strategy is shown on Figure S5.

Statistical processing of the obtained results and plotting were performed using the R-studio program (language version R 4.0.0). As part of the data pre-processing, background values (unstimulated samples) were subtracted from the values of the T-cell response level obtained after stimulation. Next, the analyzed parameters were compared at various time points after vaccination (day 30, 51, and 90) with baseline values (day 0) using a paired Mann–Whitney test. To adjust for multiple comparisons of several groups with one, the Holm correction was used.

ELISpot (Enzyme-Linked Immunosorbent Spot): PBMCs were isolated from whole blood samples and stimulated according to the manufacturer’s instructions. Each sample was tested with a panel of antigens 1—peptides of the S-protein (Spike) and panel of antigens 2—peptides of proteins N, M, ORF3a, and ORF7a (as a control) of SARS-CoV-2. Further controls included negative control to detect non-specific cell activation and positive control for confirmation of PBMC functionality. Staining was performed after 24 h of incubation according to the manufacturer’s instructions. Spot counting (SFU) was performed with an ImmunoSpot^®^ S6 analyzer (CTL).

### 2.6. Primary Outcomes

The primary outcomes related to safety and tolerability were the proportion of the subjects with any adverse event (AE) and the proportion of the subjects with any serious adverse event (SAE), both occurring within 50 days after the first administration of the study drug/placebo.

The first primary outcome related to immunogenicity and efficacy was the proportion of the volunteers with an at least 4-fold increase in the level of specific humoral immune response (titers of total anti-SARS-CoV-2 antibodies assessed by ELISA) 21 days after the second administration of the study drug/placebo and 90 ± 5 days after the first administration of the study drug/placebo. The second primary outcome was the proportion of the volunteers with a specific humoral protective immune response (assessed by SARS-CoV-2 Surrogate Virus Neutralization Test) 21 days after the second administration of study drug/placebo and 90 ± 5 days after the first administration of study drug/placebo.

### 2.7. Secondary Outcomes

#### 2.7.1. Safety Outcomes

The proportion of volunteers with at least one COVID-19 symptom (fever, chills, dyspnoea, difficulty breathing, cough, sore throat, fatigue, muscle pain, loss or decrease in taste and/or smell, nasal congestion, runny nose, headache, nausea, vomiting, diarrhea), or with a moderate, severe, extremely severe, or lethal course of COVID-19, and a PCR-confirmed SARS-CoV-2 infection from the 7th day after the second administration of the study drug/placebo till the 90 ± 5 days after the first dose of study drug/placebo was the first secondary safety outcome.

The proportion of volunteers who experienced immediate side effects (allergic reactions) within 2 h after injection of the study drug or placebo and who experienced local severe (>grade 3) post-vaccination reactions, or systemic and severe (>grade 3) side effects within 7 days after receiving the study drug or placebo was the second secondary safety outcome.

The proportion of volunteers with any AEs within 90 ± 5 days after the first dose of the study drug/placebo or with adverse events of special interest (adverse reactions that require medical attention, newly developed chronic diseases), or with SAE’s within 50 and 90 ± 5 days after the first dose of the study drug/placebo was the third secondary safety outcome.

The proportion of volunteers who prematurely terminated their participation in the study due to the development of AE’s or SAE’s associated with the use of the study drug within 50 and 90 ± 5 days after the first dose of the study drug/placebo was the fourth secondary safety outcome.

#### 2.7.2. Immunogenicity Outcomes

The proportion of volunteers with an at least a 4-fold increase in the titers of specific anti-SARS-CoV-2 IgG on day 21 after the second administration and 90 ± 5 days after the first dose of the study drug/placebo was the first secondary immunogenicity outcome.

The proportion of volunteers tested positive for the presence of anti-SARS-CoV-2 neutralizingantibodies within 51 days after the first administration of study drug/placebo was the second secondary immunogenicity outcome.

Geometric mean titers of anti-SARS-CoV-2 total and IgG antibodies on day 21 after the second administration and 90 ± 5 days after the first dose of the study drug/placebo was the third secondary immunogenicity outcome.

The proportion of volunteers from Stage I with a specific anti-SARS-CoV-2 cell-mediated immune response on day 21 after the second administration and 90 ± 5 days after the first dose of the study drug/placebo was the fourth secondary immunogenicity outcome.

### 2.8. Statistical Analysis

The calculated descriptive statistics parameters for the quantitative variables were the following: arithmetic mean (M), standard deviation (SD), 95% confidence interval (CI) of the mean, lower (LL CI) and upper (UL CI) limits, median (Me), and interquartile range (IQR). The number of observations (N), minimum (Min), and maximum (Max) were also calculated. Arithmetic mean (M), standard deviation (SD), LL CI, and UL CI were not reported as the data not following the normal distribution law. The distribution of the qualitative variables was described using proportion (W), its standard error (SE), and the 95% CI for the proportion.

The Mann–Whitney U-test or Dunn test was used to compare two independent samples described by quantitative variables, while t-test was used for normally distributed data. To compare several independent samples (more than two) described by quantitative variables, Kruskal–Wallis ANOVA, while one-way ANOVA was used for normally distributed data. When statistically significant differences between all groups were observed, the post hoc nonparametric Dunn test was applied to identify differences between groups. For intergroup comparison of qualitative indicators (proportions), a two-tailed Fisher exact test or Pearson’s goodness-of-fit test (χ2 test) was performed depending on the number of observations. For variables with three categories, proportion intergroup pairwise analysis was performed using a two-sample z-test for qualitative data at the achieved significance level *p* ≤ 0.05 in the χ2 test (after checking the assumptions).

Statistical analysis of the data obtained in this clinical study was carried out using software packages R 3.6.3, Statistica 10.0 (Statsoft, Tulsa, Oklahoma, USA), Microsoft Excel 2013 (Microsoft, Redmond, Washington, USA) with add-ons AtteStat, XLStat.

### 2.9. Role of the Funding Source

LLC Betuvax sponsored and designed the trial, provided the study drug, and managed all trial operations. The sponsors collaborated with the Contract Research Organization (CRO) “Clinical Excellence Group” (CEG BIO) to coordinate interactions with regulatory authorities and manage clinical site operations. The data was collected by the clinical site research staff, managed by a blinded CRO Data Management Team which was monitored by a CRO, and controlled by the Sponsor and an Independent Data Monitoring Committee (IDMC). The interim data analysis was performed after the primary data was reviewed and the interim database got locked. The work in preparation of this manuscript was performed by the authors of the study and the decision to submit the manuscript for publication was made by all the authors.

## 3. Results

### 3.1. Study Design

**Figure 1:**
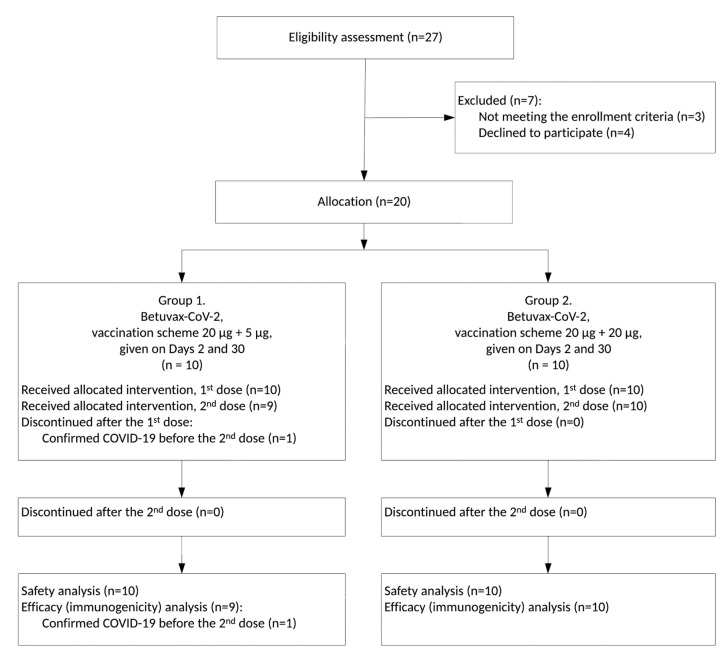
CONSORT Flow Diagram for the Clinical Trial, Stage I (preliminary version, pertains to the period of 3 weeks after the 2nd dose of the “Betuvax-CoV-2” vaccine).

**Figure 2:**
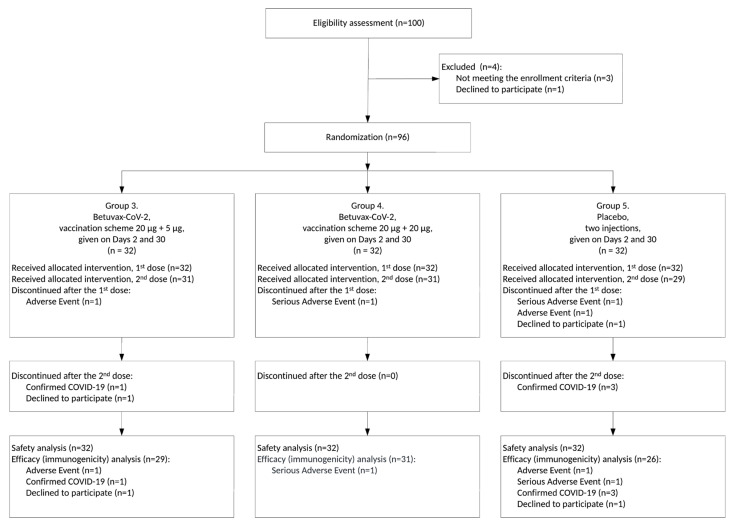
CONSORT Flow Diagram for the Clinical Trial, Stage II (preliminary version, pertains to the period of 3 weeks after the 2nd dose of the “Betuvax-CoV-2” vaccine/placebo).

Between 12 October and 23 December 2021, a total of 127 healthy volunteers, age 18–58, were screened for meeting inclusion and not meeting exclusion criteria (Table S1). Eventually, 20 and 96 participants were enrolled in Stages I and II, respectively (Figure 1 and Figure 2, Figures S1–S3).

While the interim data analysis was performed, 11 healthy volunteers ended their participation in the study ahead of the schedule: six before the second injection and five after the second. In G1 (*n* = 10), one volunteer developed COVID-19 after the first administration of the vaccine (Visit 4). In G3 (*n* = 32), 1 volunteer developed the AE “10014893 Enterocolitis” after the first administration of the drug (Visit 4), 1 volunteer developed COVID-19 after the second administration of the drug (Visit 16), and 1 volunteer withdrew from the study after the second administration of the drug (Visit 18). In G4 (*n* = 32), 1 volunteer developed the SAE “10044008 Tonsillitis” after the first administration of the drug (Visit 9). In G5 (*n* = 32), 1 volunteer developed the SAE “10015037 Epilepsy” (epileptic seizure) after the first placebo injection and one day before the second placebo injection (Visit 10), one volunteer developed the AE “10017888 Gastroenteritis” after the first placebo injection (Visit 9), three volunteers developed COVID-19 after the second placebo injection (within a 3-week period), and one volunteer declined to participate in the study after the first injection of the placebo (Visit 4). Severe adverse events such as tonsillitis and epilepsy were marked as “unlikely” (no association with vaccination), and enterocolitis and gastroenteritis were marked as “doubtful,” so the outcome in each of these cases was specified as “recovery without consequences” (Table S2).

Baseline characteristics of the participants are shown in Table 1 and Table 2. The mean age was 34.6 (SD = ±9.8), all volunteers being older than 18. BMI was in the range of 18.5–30.0 kg/m^2^. The volunteers in all groups recruited for the study met the enrollment criteria defined in the protocol. Comparative assessment of the demographic and anthropometric characteristics of volunteers did not reveal any initial statistically significant differences between the groups.

### 3.2. Safety and Tolerability

The results of the interim data analysis until Visit 18 of Stages I and II (for the period of 3 weeks after the 2nd dose of the vaccine/placebo) showed a favorable safety profile and good tolerability of the “Betuvax-CoV-2” recombinant vaccine.

During the study, the following total of 49 AE’s were registered; 13 AE’s in the Stage I (10 in G1 and 3 in G2) and 36 AE’s in Stage II (17 in G3, 4 in G4, and 15 in G5) with clinical manifestations or changes in laboratory parameters exclusively (Table S2).

In G1, 10 out of 10 AE’s (100.0%) were classified as “mild”. In 5 out of 10 cases (50.0%) the causal association with administration of the vaccine was assessed as “probable” in 4 cases (40.0%) as “possible” and in 1 case (10.0%) as “unlikely (no association).” No actions taken at the onset of AE’s in 9 out of 10 cases (90.0%) were indicated as “no action taken” and in 1 case (10.0%) the action was indicated as “symptomatic treatment”. The outcome in 7 out of 10 cases (70.0%) was “recovery without consequences” and in 3 cases (30.0%) was “ongoing AE’s”.

In G2, 3 out of 3 AE’s (100.0%) were considered “mild”. In 2 cases (66.7%), the causal association with administration of the vaccine was assessed as “probable” and in 1 (33.3%) as “doubtful”. All 3 AE’s cases (100.0%) were labeled with “no action taken”. The outcome of 3 cases (100.0%) was “recovery without consequences”. AE’s were assessed with laboratory and instrumental findings in all 3 cases (100.0%).

In G3, 15 out of 17 AE’s (88.2%) were classified as “mild” and 2 out of 17 (11.8%) as “moderate”. Two cases (11.8%) were assessed as “probable”, 11 cases (64.7%) as “possible” and 4 (23.5%) as “doubtful”. No actions taken at the onset of AE’s in 15 out of 17 cases (88.2%) were indicated as with “no action taken”, while in 2 cases (11.8%) as “symptomatic treatment”. The outcome in 15 out of 17 cases (88.2%) was “recovery without consequences”, in 1 case (5.9%) was “unknown”, while in 1 case (5.9%), the outcome was undetermined by the time of the start of the interim data analysis.

In G4, 3 out of 4 AE’s (75.0%) were classified as “mild” and 1 (25.0%) as “moderate”. Association of the three cases (75.0%) with administration of the vaccine was assessed as “doubtful” and 1 case (25.0%) as “unlikely” (no association). No actions taken in the onset of AE’s in 2 out of 4 cases (50.0%) were indicated as “no action taken” and in 2 cases the actions were indicated (50.0%) as “symptomatic treatment”. The outcome in 3 out of 4 cases (75.0%) was “recovery without consequences”, and in 1 case (25.0%) was “AE status without change”. In terms of severity, 1 out of 4 cases (25.0%) was classified as SAE with “unlikely” association with the administration of the vaccine, and 3 of 4 cases (75.0%) as “non-SAE’s”.

In G5 (placebo group), 10 out of 15 AEs (66.7%) were categorized as “mild”, and 5 (33.3%) as “moderate”. The causal relationship to the placebo was assessed as “possible” in 10 out of 15 cases (66.7%), “doubtful” in 4 cases (26.7%) and “unlikely” (no association) in 1 case (6.7%). Actions taken upon the occurrence of AE’s in 11 out of 15 cases (73.3%) were classified as “no action taken”. Four out of 15 cases (26.7%) resulted in “symptomatic treatment”. The outcome in 15 out of 15 cases (100.0%) was “recovery without consequences”. By severity, 1 case (6.7%) was considered SAE, and 14 out of 15 cases (93.3%) non-SAE’s (Table S2).

The proportion of volunteers with any AE’s developing within 50 days of the first administration of the vaccine was higher for G1 (20 + 5 µg dosing regimen) in comparison with G2 (20 + 20 μg) in Stage I (100% vs. 30%, *p* = 0.003, Fisher exact test). On Stage II, the proportion was significantly lower in G4 vs. G3 (9.4% vs. 53.1%, *p* < 0.001, Fisher exact test), and in G4 vs. G5 (9.4% vs. 46.9%, *p* = 0.002, Fisher exact test). The second primary safety outcome, the proportion of volunteers with SAE’s developing within 50 days of the first administration of study drug/placebo, showed no statistically significant difference between all the study groups. Additionally, no SAE’s related to vaccination were reported during the trial.

The proportion of the volunteers with general post-vaccination reactions developed within 7 days after the first and the second injections in G1 vs. G2 (0% vs. 0%) and in G3 vs. G4 vs. G5 (0% vs. 3.1% vs. 3.1%) did not significantly differ between groups. No AE’s of the two following types were reported by an investigator or a volunteer during the clinical trial; immediate adverse events (allergic reactions) occurred within 2 h after administration of the first and second injection of the study drug/placebo; local and systemic reactions (>grade 3) occurring within 7 days after administration of the first and second injection of the study drug/placebo.

The frequency of all studied AE’s between G1 and G2 during the entire period of the trial did not differ significantly. Similar results were obtained for Stage II. In terms of the frequency of early termination of participation in the study due to the occurrence of AEs, the groups did not significantly differ from each other.

According to the safety criteria, the absence of significant intergroup differences, features and distribution of the observed AE’s, the absence of clinically significant intergroup differences in terms of vital signs (body temperature, blood pressure, heart and respiratory rates), instrumental parameters (ECG), and laboratory tests (clinical blood test, biochemical blood test and urinalysis) demonstrated good safety profile and tolerability of the vaccine.

### 3.3. Humoral Immune Response

The results of the interim data analysis after all participants of the Stage I completed Visit 19 and all participants of the Stage II completed Visit 18 (or after the early termination of the participation in the study before the aforementioned Visits) demonstrated high immunological effectiveness (immunogenicity) of the “Betuvax-CoV-2” recombinant vaccine.

**Figure 3:**
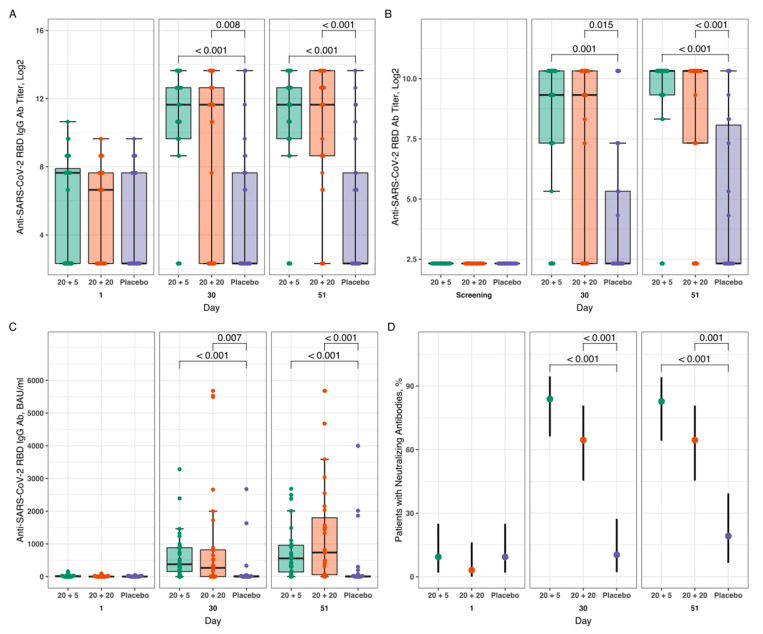
(**A**) Specific IgG, (**B**) total antibodies titers, and (**C**) neutralizing antibodies (BAU/mL) against the SARS-CoV-2 RBD-antigen over time after vaccination with “Betuvax-CoV-2” at 20 + 5 µg (G3), 20 + 20 µg (G4) and in placebo group (G5). (**D**) Proportions of the participants with anti-SARS-CoV-2 neutralizing antibodies over time after the administration of the “Betuvax-CoV-2” at 20 + 5 µg (G3), 20 + 20 µg (G4) and in placebo group (G5).

Groups G1 (20 + 5 µg dosing regimen) and G2 (20 + 20 µg) of the Stage I were not significantly different from each other in all immunogenicity and efficacy parameters assessed 21 days following the second dose of the study drug and 90 ± 5 days after the first dose of the study drug. In both groups, a marked increase (from Visits 0/1 to 19) of the humoral anti-SARS-CoV-2 immune response was observed (Figure S4). At Stage II of the study, G3 (20 + 5 µg) as compared to G5 (placebo) and G4 (20 + 20 µg) as compared to G5 (placebo) demonstrated statistically significant differences in most of the assessed immunogenicity parameters 21 days after the second dose of the study drug/placebo. The dynamics of total, IgG and neutralizing antibodies against the SARS-CoV-2 RBD-antigen in volunteers of the groups G3, G4, and G5 on day 1 (screening), day 30 (Visit 11), and day 51 (Visit 18) after the first vaccine administration are shown on Figure 3.

Geometric Mean Titers (GMTs) of anti-SARS-CoV-2 IgG antibodies specific to the RBD antigen 21 days after administration of the second dose of study drug/placebo were as follows: 1479.1 and 1271.2 vs. 46.8 (G3 and G4 vs. G5), respectively (95% CI for difference in mean log-transformed titers in G3 and G5 (2.91; 7.06); 95% CI for difference in mean log-transformed titers in G4 and G5 (2.68; 6.85)) (Figure 3A). In both test groups in Stage II (G3 and G4), proportions of participants with at least a 4-fold or more increase of the anti-SARS-CoV-2 IgG antibody titers against the RBD antigen 21 days after administration of the second dose of study drug/placebo were significantly higher compared with those in G5 at 2.6 (*p* < 0.001, χ2 test) and 2.7 (*p* < 0.001, χ2 test), respectively: 79.3% and 83.9% vs. 30.8% (G3 and G4 vs. G5).

GMTs of total anti-SARS-CoV-2 antibodies against the RBD antigen 21 days after administration of the second dose of test drug/placebo were as follows: 581.6 and 292.6 vs. 25.4 (G3 and G4 vs. G5), respectively (95% CI for difference in mean log-transformed titers in G3 and G5 (2.93; 6.11); 95% CI for difference in mean log-transformed titers in G4 and G5 (1.76; 5.29) (Figure 3B). In G3 and G4, proportions of participants with increased levels of a specific humoral immune response (anti-SARS-CoV-2 total antibody titers against the RBD antigen) by at least 4-fold or more 21 days after administration of the second dose of study drug/placebo were statistically greater compared to G5 (placebo group) by a factor of 2.33 (*p* < 0.001, χ2 test) and 2.01 (*p* < 0.001, χ2 test), respectively: 89.7% and 77.4% vs. 38.5%.

It was also found that administration of the “Betuvax-CoV-2” according to 20 + 5 µg (G3) and 20 + 20 µg (G4) regimens compared to placebo (G5) resulted in a significant increase in Median Concentration (MCs) of IgG-antibodies against the RBD antigen of SARS-CoV-2 21 days after the second dose of the study drug (MCs in G3 vs. G5: 556.88 vs. 2.96 BAU/mL, *p* = 0.001, Dunn test; MCs in G4 vs. G5: 736.71 vs. 2.96 BAU/mL, *p* < 0.001, Dunn test) (Figure 3C).

In G3 and G4, proportions of participants with the presence of anti-SARS-CoV-2 neutralizing antibodies 21 days after administration of the second dose of test drug/placebo were significantly higher comparing to G5 in 4.3 (*p* < 0.001, χ2 test) and 3.4 (*p* = 0.001, χ2 test) times, respectively: 82.8% and 64.5% vs. 19.2% (Figure 3D).

At the same time, G3 and G4 did not differ significantly when evaluating according to the above immunogenicity endpoints, suggesting that the absence of significant differences in the humoral immune response to the intramuscular administration of the “Betuvax-CoV-2” at 20 + 5 µg and 20 + 20 µg dosing regimens.

### 3.4. Cell-Mediated Immune Response

**Figure 4:**
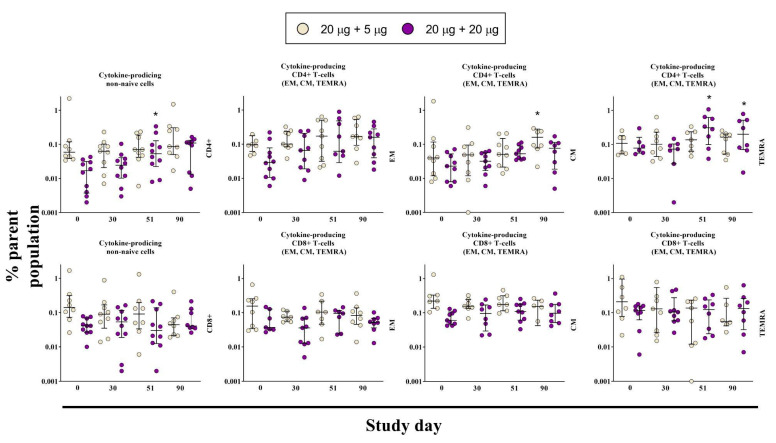
The post-vaccination antigen-specific cell-mediated immune response in the non-naive CD4+ and CD8+ T-cell populations on days 0, 30, 51, and 90. Cells were considered positive if at least one cytokine out of IFN-γ, IL-2, and TNF-α was expressed. Comparisons were made to day 0 in the corresponding group. *p* ≤ 0.05 is indicated with *.

In order to study a cell-mediated immune response, samples from G1 and G2 were analyzed by flow cytometry at several time points during the study. Cryopreserved PBMCs samples were collected before vaccination (day 0, Visit 0) and at day 30 (Visit 11), 51 (Visit 18) and 90 (Visit 19) after the first administration (Figure 4). Using the CD45RA and CCR7 markers, populations of central memory (CM) and effector memory (EM) T-cells, as well as terminally differentiated effector T-cells (TEMRA) were analyzed. The immune response was assessed on the basis of the intracellular production of cytokines IFN-γ, IL-2, and TNF-α in various populations of T-cells.

Total population of EM, CM, and TEMRA of antigen-specific cytokine-producing CD4+ T-lymphocytes in G2 volunteers (20 μg + 20 μg) was increased after the second administration of the vaccine on day 51 and remained higher than day 0 until day 90, however the difference was not significant (Figure 4). The population of CM cells was increased in the G1 group on day 90 (20 μg + 5 μg). The TEMRA population also increased after the second administration of the vaccine on day 51, and remained the same on day 90. There were no differences detected in either total number of antigen-specific cytokine-producing CD8+ T-cells or when assessed by individual subpopulations (Figure 4). Meanwhile, according to the ELISpot assay conducted for groups G3, G4, and G5, there was no significant difference between the vaccine groups and placebo on the 21st day following the second administration of the drug/placebo (Figure S6). This could be due to sample preparation for the assay, specifically freezing of the samples, as well as due to some hidden infections in the placebo group. Nevertheless, the proportion of volunteers with a specific anti-SARS-CoV-2 cell-mediated immune response was a secondary immunogenicity criterion and we are planning to conduct additional analysis of the cell-mediated immunity induced by the vaccine with some modifications of the sample preparation procedure.

## 4. Discussion

During this trial the “Betuvax-CoV-2” vaccine has shown a favorable safety profile in comparison with vaccines that already reached the market, such as BNT162b2/Comirnaty (Pfizer/Biontech, Mainz, Germany), mRNA-1273/Spikevax (Moderna, Cambridge, MA, USA), Vaxzevria/AZD1222/ChAdOx1 (AstraZeneca/Oxford University), or Jcovden (Janssen, Beerse, Belgium). The vast majority of the observed AEs of these vaccines represent the local injection reactions, such as pain, redness, and swelling. Frequently reported systemic AEs are fever, headache, fatigue, and myalgia [5,6,7,8,9,10]. With“Betuvax-CoV-2”, there were no statistically significant differences between the vaccine groups and the placebo in terms of local and systemic post vaccination reactions.

In addition, adverse events of special interest (AESI), such as shortness of breath, arrhythmias, myocarditis, pulmonary embolism, and apoplexy, associated with BNT162b2/Comirnaty (Pfizer/Biontech, Mainz, Germany), mRNA-1273/Spikevax (Moderna, Cambridge, MA, USA), Vaxzevria/AZD1222/ChAdOx1 (AstraZeneca/Oxford University), and Jcovden (Janssen, Beerse, Belgium) were also reported [11]. Recent reports have shown that the rare incidence of venous thrombosis could be associated with adenovirus vaccine Vaxzevria (AstraZeneca/Oxford University) [12]. Severe side effects of mRNA vaccines were also registered and included anaphylaxis, peripheral facial paralysis (Bell’s palsy), paroxysmal ventricular arrhythmia, and leg paresthesias [13]. In the “Betuvax-CoV-2” first clinical trial, no SAEs related to the vaccination were observed. Thus, the “Betuvax-CoV-2” vaccine has a good safety profile suitable for mass immunization. However, a long-term assessment on the bigger number of participants, including children and elderly, has to be conducted as well.

Detection of neutralizing antibodies, which provide necessary protection against SARS-CoV-2, plays a significant role in assessing the immune status of an individual after vaccination. The gold standard for the quantification of neutralizing antibodies is the Plaque Reduction Neutralization Test (PRNT). However, it requires at least a Safety Level 3 (BSL-3) laboratory, 2−5 days for the testing itself and a significant financial outlay. An alternative to PRNT is the surrogate Virus Neutralization Test (sVNT) (GenScript, Piscataway, New Jersey, US) that was used in this study. There is a good correlation between PRNT values and the binding inhibition values obtained with GenScript’s sVNT [14,15]. Thus, on day 21 after the second administration of the vaccine, the proportion of volunteers with neutralizing antibodies was 4.3-fold (G3) and 3.4-fold (G4) higher compared to the placebo group (G5).

Another test, “SARS-CoV-2 IgG II Quant test system” (Abbott Laboratories, Chicago, Illinois, United States), for the detection of RBD-binding antibodies shows a good correlation between the sVNT values and is widely used for the assessment of immunity against SARS-CoV-2 [16]. For instance, the median level of the anti-RBD IgG antibodies up to 24 weeks after the prime-boost vaccination were around 600–2500 BAU/mL for mRNA-based vaccines, such as Comirnaty (Pfizer/Biontech, Mainz, Germany) and Spikevax (Moderna, Cambridge, MA, USA) [17,18,19]; for adenovirus-based vaccines as Vaxzevria (AstraZeneca/Oxford University) and Sputnik V (Gamaleya National Center of Epidemiology and Microbiology, Moscow, Russia)—around 200 BAU/mL [19,20,21]; for inactivated vaccine CoronaVac (Beijing, People’s Republic of China) this value was around 35 BAU/mL [22]. Therefore, compared to other common vaccines, the immunogenicity of the “Betuvax-CoV-2” at 20+20 μg dosing regimen, producing around 750 BAU/mL antibodies (measured by the Abbott test), is presumably on the similar level as with the mRNA-vaccines (Comirnaty, Spikevax) but has better RBD-based immunogenicity than inactivated (CoronaVac) and vector vaccines (Vaxzevria, Sputnik V).

Assessing the correlation between protectivity from the SARS-CoV-2 infection and the neutralizing antibody levels is a relevant question, but the optimal antibody titer has not been precisely established at this time. In the COVE trial, an RBD titer of 775 BAU/mL correlated with 90% protection from symptomatic infection with the wild type SARS-CoV-2 [23], while in another study [24], this threshold was above 141 BAU/mL. In the COV002 trial, an RBD titer of 506 BAU/mL was associated with an 80% protection from the symptomatic infection of the Alpha variant of the SARS-CoV-2 [25]. In the recent study [26], at least 6000 BAU/mL was required for the effective protection against Omicron strains. Therefore, the booster vaccination is required, being able to lead to higher levels of antibodies in comparison with a one-time vaccination of naive participants, and also antigen adaptation to the Omicron strains. The updated version of “Betuvax-CoV-2” vaccine as the booster administration would be evaluated during the phase III/IV clinical trial as well as its clinical efficacy.

Other limitations of this study are following: the inability to control the SARS-CoV-2 exposure factors (possible undetected by PCR/symptomatically infections); the lack of data to assess potential differences in vaccine efficacy in different sex and age groups; Wuhan-based vaccine study was conducted during the emergence of the new SARS-CoV-2 strains which precluded the assessment of the vaccine efficacy.

## 5. Conclusions

The preliminary results of phase I/II clinical trial indicated that the protein-subunit vaccine against SARS-CoV-2 “Betuvax-CoV-2” has a sufficient safety profile and immunogenicity. This study showed a marked increase in the specific anti-SARS-CoV-2 humoral response after the administration of the vaccine. Based on these results, a one-dose (20 µg) and a two-dose (20 + 20 µg on days 0 and 28) intramuscular immunization regimens would be further tested for efficacy in phases I/II (booster) and III clinical trials, respectively.

## Figures and Tables

**Table 1:** Baseline characteristics of the study participants in Stage I (G1 and G2).

	Group	N	M ± SD	95% CI	Min–Max	Me	IQR	*p*
Weight, kg	G1	10	64.2 ± 10.6	56.6–71.8	48.0–79.0	63.0	13.0	0.571 ^2^
	G2	10	67.3 ± 13.3	57.8–76.8	50.0–90.0	65.0	20.0	
Age, years	G1	10	-	-	26.1–57.5	32.2	6.9	0.853 ^1^
	G2	10	-	-	18.3–51.4	35.6	16.2	
BMI, kg/m	G1	10	-	-	18.8–29.4	20.6	5.2	0.853 ^1^
	G2	10	-	-	18.6–29.8	20.4	4.6	
Height, cm	G1	10	170.5 ± 7.8	164.9–176.1	160.0–186.0	170.0	12.0	0.375 ^2^
	G2	10	174.1 ± 9.8	167.1–181.1	160.0–188.0	173.5	17.0	

^1^ Mann–Whitney test. ^2^ t-test for independent samples. M ± SD—Mean ± Standard deviation, CI—Confidence interval, Me—Median, IQR—Interquartile range (Q3–Q1).

**Table 2:** Baseline characteristics of the study participants in Stage II (G1, G2, and G3).

	Group	N	M ± SD	95% CI	Min–Max	Me	IQR	*p*
Weight, kg	G3	32	-	-	55.3–96.0	71.9	15.1	0.157 ^1^
	G4	32	-	-	50.0–100.1	65.1	12.0	
	G5	32	-	-	52.0–92.3	67.1	16.7	
Age, years	G3	32	-	-	19.6–53.2	31.8	12.9	0.709 ^1^
	G4	32	-	-	19.4–58.1	28.7	14.0	
	G5	32	-	-	20.8–58.7	29.9	11.2	
BMI, kg/m	G3	32	23.6 ± 2.3	24.5–22.8	18.8–28.3	23.4	3.2	0.660 ^2^
	G4	32	23.7 ± 2.4	24.6–22.9	19.6–28.8	23.8	4.1	
	G5	32	23.2 ± 3.0	24.2–22.1	18.6–29.3	22.7	4.3	
Height, cm	G3	32	-	-	155.0–190.0	174.0	9.5	0.590 ^1^
	G4	32	-	-	153.0–195.0	167.5	12.0	
	G5	32	-	-	158.0–190.0	169.5	13.5	

^1^ Kruskal–Wallis test. ^2^ One-Way ANOVA test. M ± SD—Mean ± Standard deviation, CI—Confidence interval, Me—Median, IQR—Interquartile range (Q3–Q1).

## Data Availability

The datasets generated or analyzed during this study are available from the corresponding author on a reasonable request.

## Supplementary Materials

The following supporting information can be downloaded at: https://www.mdpi.com/article/10.3390/vaccines11020326/s1, Figure S1: Design of the study Protocol Betuvax-CoV-2.2021.CT1-2.RUS, Stage I, Part 1; Figure S2: Design of the study Protocol Betuvax-CoV-2.2021.CT1-2.RUS, Stage I, Part 2.; Figure S3: Design of the study Protocol Betuvax-CoV-2.2021.CT1-2.RUS, Stage II; Figure S4: Anti-SARS-CoV-2 IgG, total and neutralizing antibodies after vaccination with “Betuvax-CoV-2”; Figure S5: Gating strategy for assessment of anti-SARS-CoV-2 cell-mediated immune response by flow cytometry; Figure S6: Proportion of the participants with anti-SARS-CoV-2 cell-mediated immune response assessed by ELISpot assay; Table S1: Inclusion and exclusion criteria of the study participants; Table S2: Adverse Events (preliminary data).
