# Supplementary material for: Safety and Immunogenicity of Betuvax-CoV-2, an RBD-Fc-Based SARS-CoV-2 Recombinant Vaccine: Preliminary Results of the First-in-Human, Randomized, Double-Blind, Placebo-Controlled Phase I/II Clinical Trial
Source: Vaccines (Basel). 2023 Feb 1;11(2):326. doi: 10.3390/vaccines11020326 (PMC9965088; doi:10.3390/vaccines11020326)
Supplement: Supplementary file 1 [file vaccines-11-00326-s001.zip › vaccines-2077265-supplementary.pdf]

Supplementary Material

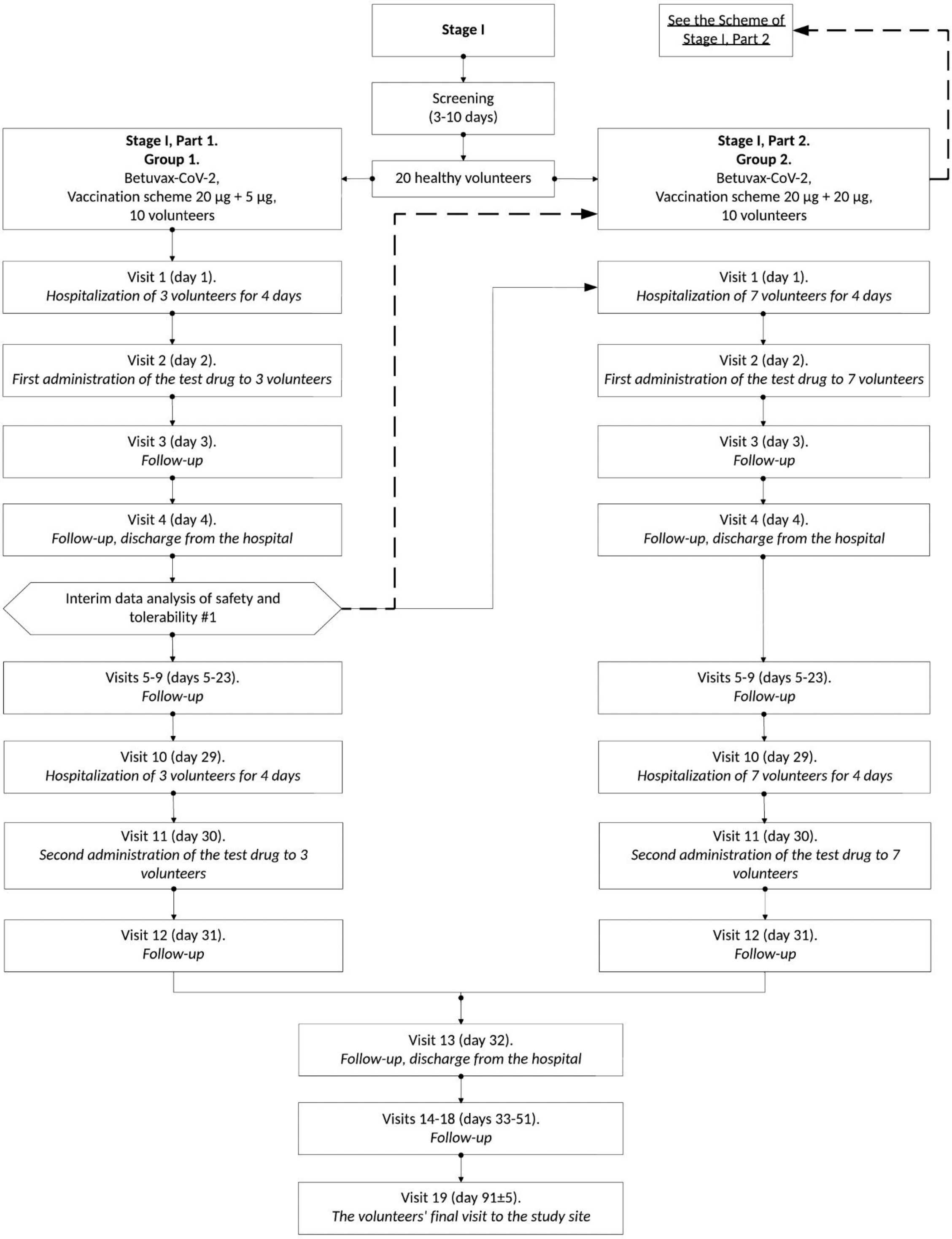

Figure S1. Design of the study Protocol Betuvax-CoV-2.2021.CT1-2.RUS, Stage I, Part 1.

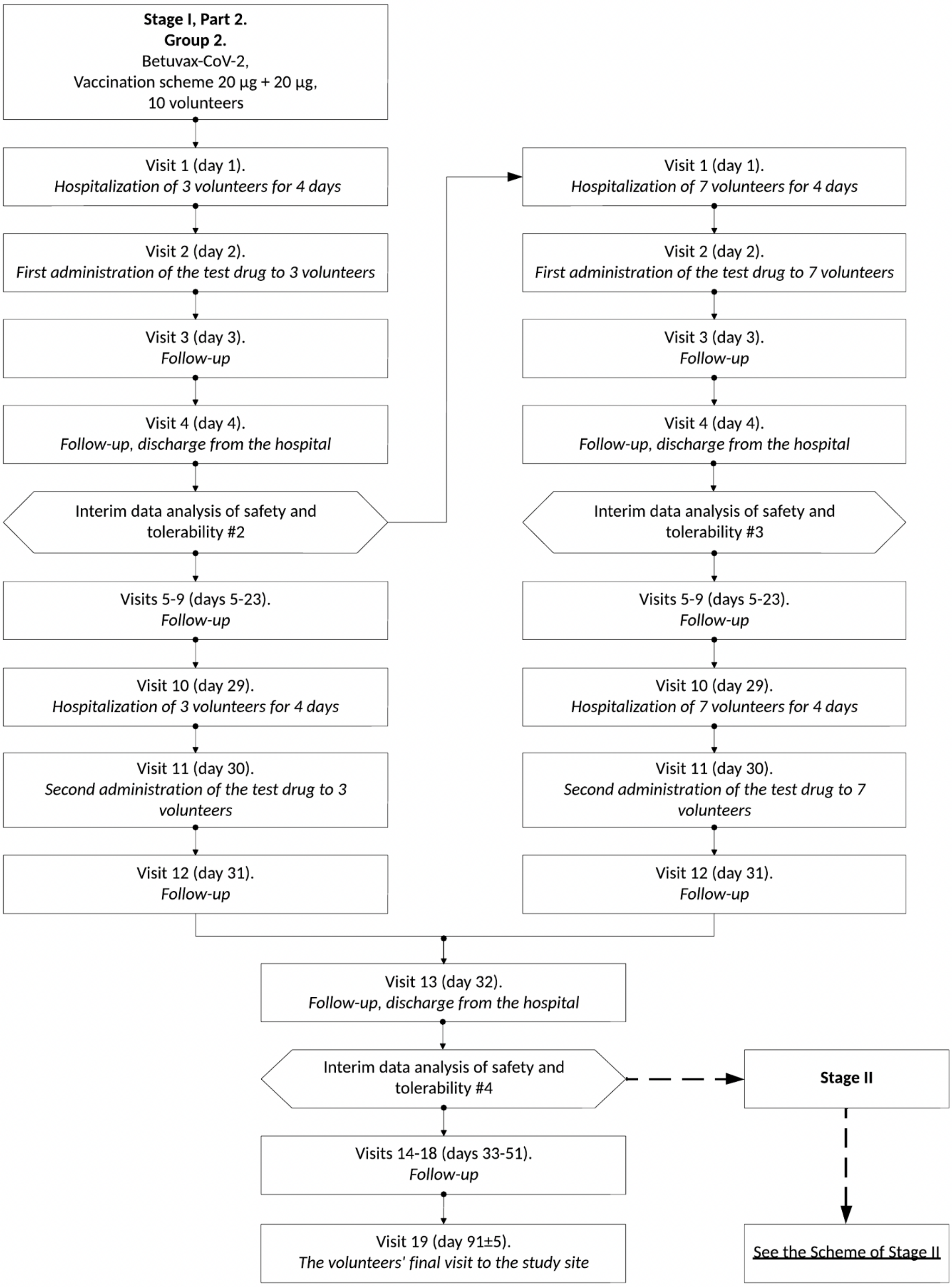

Figure S2. Design of the study Protocol Betuvax-CoV-2.2021.CT1-2.RUS, Stage I, Part 2.

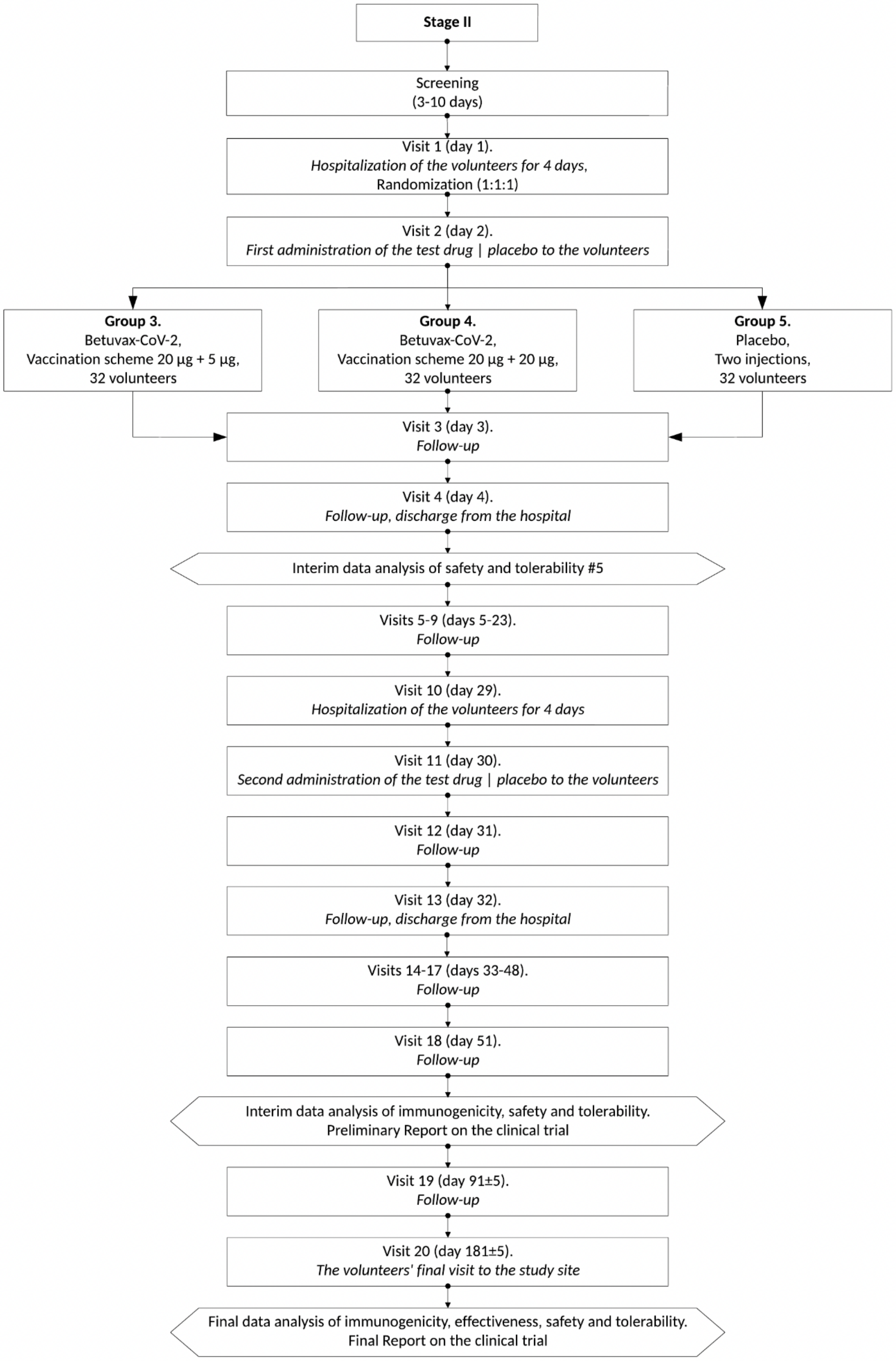

Figure S3. Design of the study Protocol Betuvax-CoV-2.2021.CT1-2.RUS, Stage II.

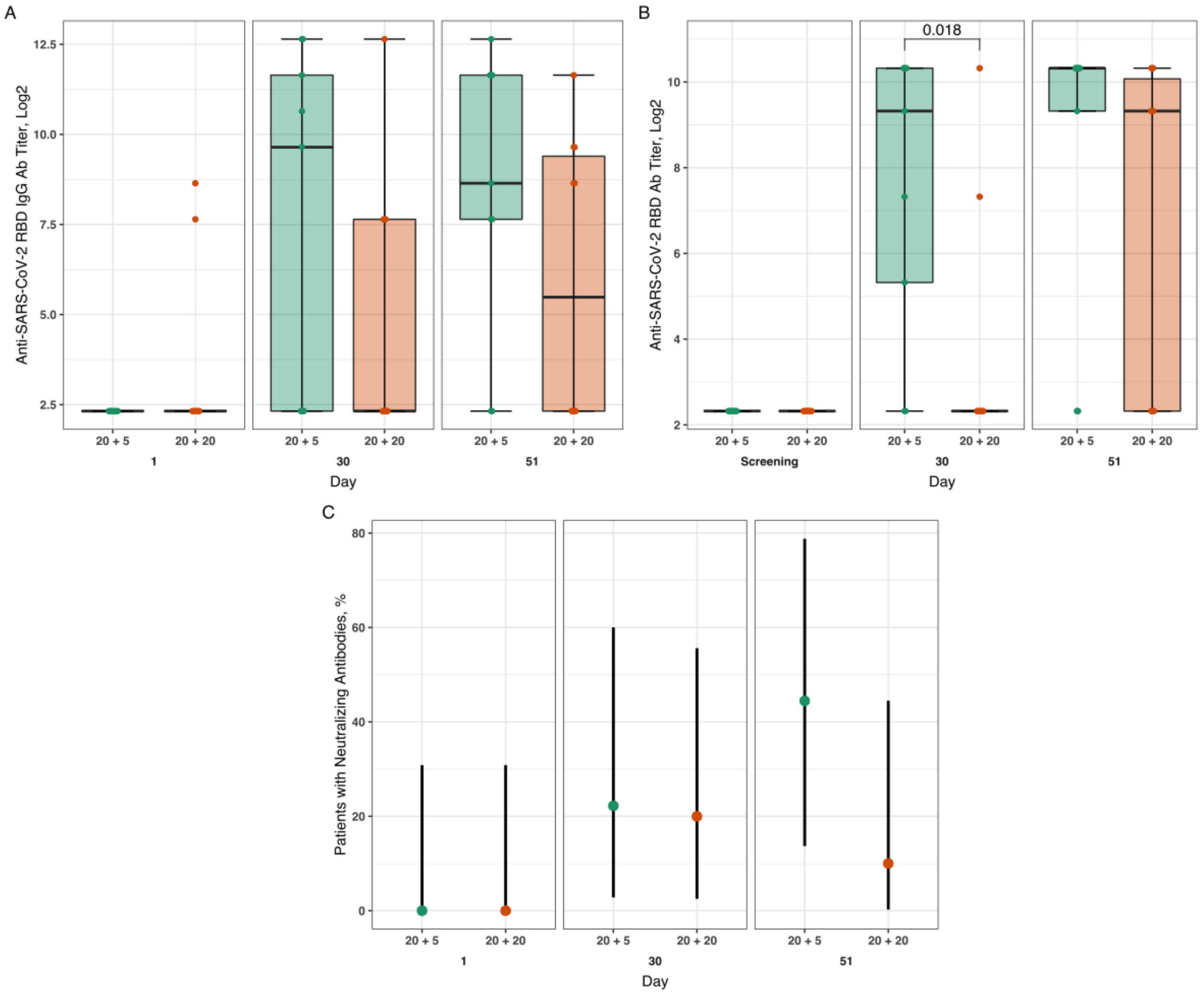

Figure S4. (A) Specific IgG and (B) total antibodies titers against the SARS-CoV-2 RBD-antigen after vaccination with "Betuvax-CoV-2" at 20 + 5 µg (G1) and 20 + 20 µg (G2). (C) Proportion of the participants with anti-SARS-CoV-2 neutralizing antibodies after vaccination with "Betuvax-CoV-2" at 20 + 5 µg (G1) and 20 + 20 µg (G2).

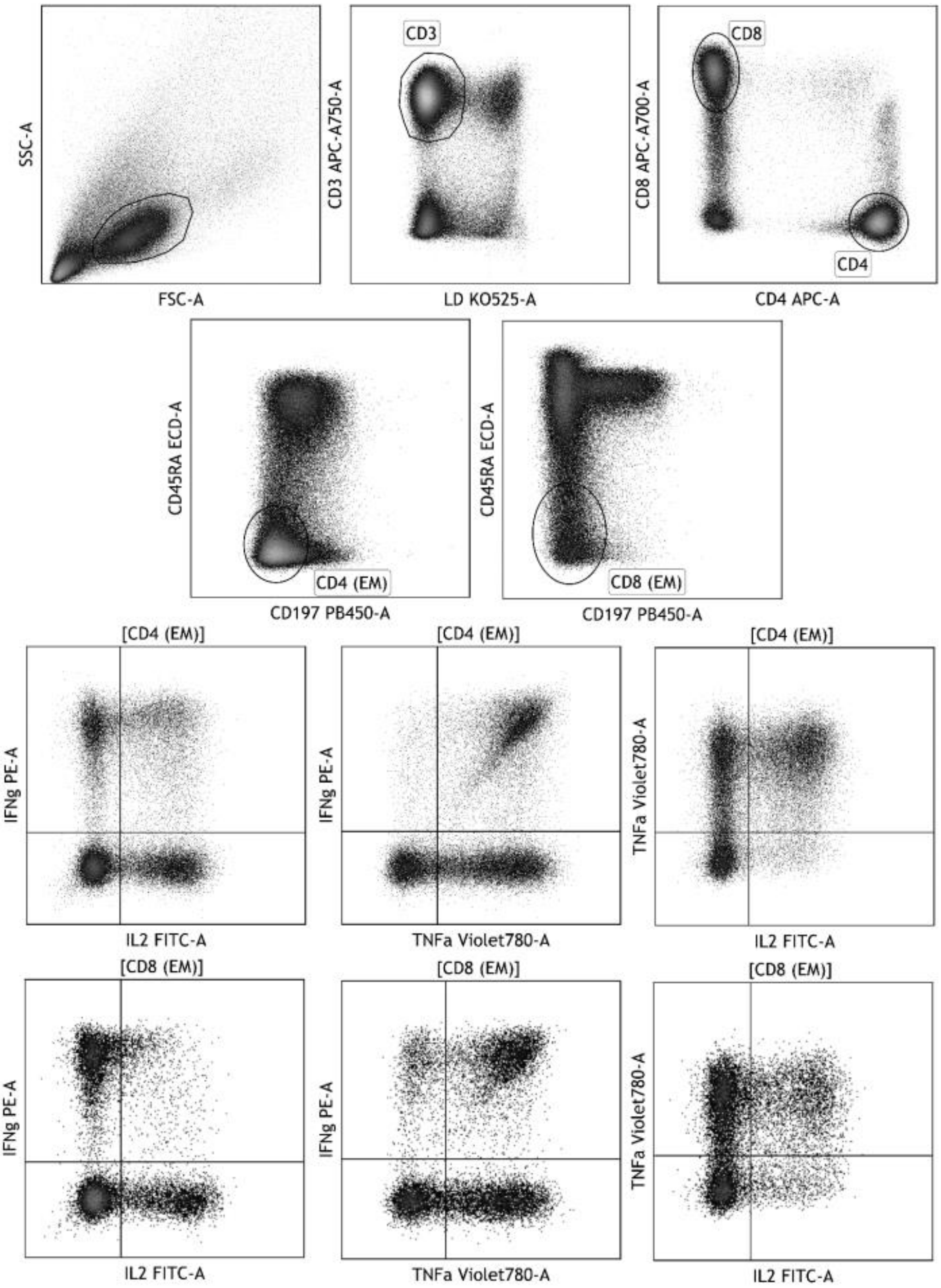

Figure S5. Gating strategy. Live and dead cells are separated using the Zombie Aqua viability marker. By the presence of the CD3 marker, a population of lymphocytes is detected, which, based on the expression of CD4 and CD8, is divided into populations of T-helpers and cytotoxic T-lymphocytes. With the help of CD45RA and CCR7 markers, populations of central (CM), effector (EM) memory T cells, as well as terminally differentiated effector T cells (TEMRA) are detected. The immune response is assessed on the basis of intracellular production of cytokines IFN-γ, IL-2, TNF-α in various populations of T cells.

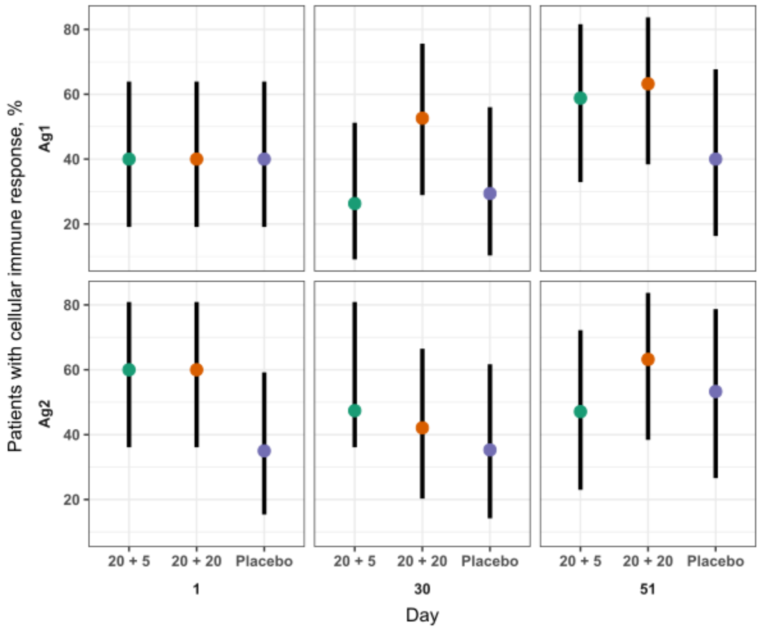

Figure S6. Proportion of the participants with specific anti-SARS-CoV-2 cell-mediated immune response assessed by ELISpot assay (panels Ag1 and Ag2) in groups G3 (20+5), G4 (20+20) and G5 (placebo).

Table S1. Inclusion and exclusion criteria of the study participants.

Inclusion criteria	Exclusion criteria
1. 18 to 60 years of age.	1. Contact with COVID-19 patients during 14 days before the start of the study.
2. Verified healthy condition according to the data of standard clinical, laboratory and instrumental examination methods.	2. PCR SARS-CoV-2 positive test.
3. For women being in an active reproductive period: Consent to adhere to appropriate methods of contraception during the entire period of the participation in the study and for 1 month after the completion of the participation in the study (appropriate methods of contraception include abstinence from sexual intercourse or any two of the following methods: an intrauterine device (intrauterine device without release and with a release of a local hormonal drug), diaphragm, spermicides, cervical caps, oral contraceptives, contraceptive sponge and/or condom); for men: Consent to adhere to appropriate dual barrier methods of contraception during the entire period of the participation in the study and 1 month after completion of the participation in the study.	3. Titer of the total specific anti-SARS-CoV-2 antibodies (ELISA) more than 1:10.
4. Body mass index (BMI): 18.5≤ BMI≤30 kg/m2.	4. Drug allergies, hereditary angioedema.
5. Negative breath alcohol test.	5. Hypersensitivity to any component of the vaccine or any excipients of Betuvax-CoV-2 or allergy to the components of the vaccine.
6. Negative laboratory blood tests for HIV, syphilis and hepatitis B and C.	6. Intolerance to any of the components or any excipients of the vaccine Betuvax-CoV-2.
	7. Allergic reaction to previous immunizations.
	8. Serious post-vaccination reactions/complications associated with previous immunizations.
	9. For women of childbearing potential - lactation period, pregnancy or suspicion of it, early postpartum period.
	10. Women in the premenopausal period (last menstrual period <1 year prior to signing informed consent) who are not surgically sterile and women who have childbearing potential but do not use or plan to use appropriate methods of contraception throughout the study and do not agree to perform a urine pregnancy test while participating in a study.
	11. Men who serve in the military by conscription.
	12. Individuals in custody in pre-trial detention centers and those serving sentences in places of deprivation of liberty.
	13. Children under 18 years of age.

7. Ability and willingness to attend all scheduled visits and undergo all procedures and examinations planned by the Protocol.<br>8. Signed and dated Informed Consent to participate in the study.	14. Chronic diseases (including oncological and autoimmune), diseases of the cardiovascular, bronchopulmonary, neuroendocrine systems, as well as the gastrointestinal tract, liver, kidneys, blood, central nervous system; surgical interventions on the gastrointestinal tract (with the exception of appendectomy).<br>15. Active tuberculosis at the time of screening (based on history and physical examination).<br>16. Mental illness, current or in history.<br>17. Decompensated neuropsychiatric diseases, including schizophrenia, multiple sclerosis, Parkinson's disease, dementia, endogenous depression, etc., which complicate the participation of a volunteer in the study.<br>18. Acute infectious from less than 3 months before the start of the study.<br>19. Acute infectious or non-infectious diseases, exacerbation of chronic diseases from less than 4 weeks before the start of the study.<br>20. Symptoms of any diseases at the time of enrollment or if less than 4 weeks have passed since recovery.<br>21. Hepatic or renal failure, currently or in history.<br>22. Current or history of oncological diseases.<br>23. Major surgery, major trauma less than 6 months prior to study initiation.<br>24. History of splenectomy.<br>25. Other comorbidities that, in the opinion of the investigator, may interfere with the evaluation of the objectives of the study.<br>26. Blood pressure: systolic blood pressure less than 100 mmHg or above 130 mmHg and diastolic blood pressure over 90 mmHg or less than 70 mmHg.<br>27. Heart rate less than 60 beats/min or more than 90 beats/min.<br>28. Deviations from the normal values according to standard clinical, laboratory (general and biochemical blood tests, urinalysis) and instrumental methods (including ECG) examination.<br>29. Long-term use (more than 14 days) of immunosuppressants, systemic glucocorticosteroids or immunomodulatory drugs during the 6 months before the start of the study.<br>30. Any vaccination within one month prior to the start of the clinical trial.<br>31. Taking medications containing immunoglobulin or blood products during the last 3 months before the start of the study.<br>32. Donation of blood (450 ml of blood or plasma and more) less than 2 months before the start of the study.<br>33. Participation in another clinical study less than 3 months before the start of the study.<br>34. Consumption of more than 10 units of alcohol (1 unit of alcohol is equivalent to 1/2 liter of beer, 200 ml of wine or 50 ml of spirits) per week or history of alcoholism, drug addiction, drug abuse.<br>35. Smoking more than 10 cigarettes a day.

	36. Special diet (for example, vegetarian, vegan, with limited salt intake) or a special lifestyle (work at night, extreme physical activity).<br>37. Positive urine test result for psychotropic and narcotic substances, psychoactive drugs (barbiturates, benzodiazepines, methadone, phencyclidine).<br>38. Unwillingness or inability to follow the recommendations and procedures prescribed by this protocol.

Table S2. Adverse Events (preliminary data).

AE #	Participant #	Group	AE Summary	PT – Preferred Term, MedDRA	SOC – System Organ Class, MedDRA	SAE	AE Outcome	AE associated with the test drug	Exclusion from the study
1	R002	G1	Increase in the absolute and relative eosinophil count (stage 1)	10014945 Increase in eosinophil count	10022891 Laboratory and instrumental data	N	Recovery without any consequences	Probable	N
2	R002	G1	Decrease in the relative number of segmented neutrophils (stage 1)	10029366 Decrease in the number of neutrophils	10022891 Laboratory and instrumental data	N	Recovery without any consequences	Probable	N
3	R002	G1	Increase in the level of total bilirubin (stage 1)	10005364 Increase in the level of bilirubin in the blood	10022891 Laboratory and instrumental data	N	Recovery without any consequences	Possible	N
4	R002	G1	Increase in the levels of transaminases (ALT and AST)	10054889 Increase in transaminase levels	10022891 Laboratory and instrumental data	N	Recovery without any consequences	Possible	N
5	R003	G1	Increase in the relative content of lymphocytes (stage 1)	10054889 Increase in the number of lymphocytes	10022891 Laboratory and instrumental data	N	In process	Probable	N
6	R003	G1	Increase in the relative content of segmented neutrophils (stage 1)	1002936 Increase in the number of neutrophils	10022891 Laboratory and instrumental data	N	In process	Probable	N
7	R004	G1	COVID-19	10084268 Coronavirus infection COVID-19	10021881 Infections and infestations	N	In process	Unlikely (no association)	Y
8	R006	G1	Decrease in the relative number of segmented neutrophils (stage 1)	10029366 Decrease in the number of neutrophils	10022891 Laboratory and instrumental data	N	Recovery without any consequences	Probable	N
9	R008	G1	Increase in body temperature (up to 37.3)	10037660 Pyrexia	10018065 General disorders and	N	Recovery without any consequences	Possible	N

					injection site reactions				
10	R008	G1	Weakness	10003549 Asthenia	10018065 General disorders and injection site reactions	N	Recovery without any consequences	Possible	N
11	R014	G2	Increase in the low-density lipoprotein levels	10024910 Increase in the low-density lipoprotein levels	10022891 Laboratory and instrumental data	N	Recovery without any consequences	Doubtful	N
12	R017	G2	Increase in the absolute and relative number of lymphocytes	10054889 Increase in the number of lymphocytes	10022891 Laboratory and instrumental data	N	Recovery without any consequences	Probable	N
13	R017	G2	Decrease in the relative number of segmented neutrophils	10029366 Decrease in the number of neutrophils	10022891 Laboratory and instrumental data	N	Recovery without any consequences	Probable	N
14	R022	G4	Lymphadenopathy	10025197 Lymphadenopathy	10005329 Blood and lymphatic system disorders	N	Status unchanged	Doubtful	N
15	R022	G4	Acute tonsillitis of moderate severity. Local lymphadenitis	10044008 Tonsillitis	10021881 Infections and infestations	Y	Recovery without any consequences	Unlikely (no association)	Y
16	R023	G5	Headache	10019211 Headache	10029205 Nervous system disorders	N	Recovery without any consequences	Possible	N
17	R040	G3	Headache	10019211 Headache	10029205 Nervous system disorders	N	Recovery without any consequences	Possible	N
18	R043	G4	Acute respiratory viral infection	10047461 Viral infection	10021881 Infections and infestations	N	Recovery without any consequences	Doubtful	N
19	R045	G5	COVID-19	10084268 Coronavirus infection COVID-19	10021881 Infections and infestations	N	Recovery without any consequences	Doubtful	Y
20	R057	G3	Chills	10008531 Chills	10018065 General disorders and injection site reactions	N	Recovery without any consequences	Probable	N
21	R057	G3	Increase in body temperature	10037660 Pyrexia	10018065 General disorders and injection site reactions	N	Recovery without any consequences	Probable	N
22	R057	G3	Headache	10019211 Headache	10029205 Nervous system disorders	N	Recovery without any consequences	Possible	N

23	R063	G4	Acute respiratory viral infection. Acute nasopharyngitis	10047461 Viral infection	10021881 Infections and infestations	N	Recovery without any consequences	Doubtful	N
24	R068	G3	Anemia	10002034 Anemia	10005329 Blood and lymphatic system disorders	N	N/A	Doubtful	N
25	R070	G5	Convulsive syndrome. Generalized convulsive seizure from 21.01.2022	10015037 Epilepsy	10029205 Nervous system disorders	Y	Recovery without any consequences	Unlikely (no association)	Y
26	R072	G3	Headache	10019211 Headache	10029205 Nervous system disorders	N	Recovery without any consequences	Possible	N
27	R075	G5	An increase in blood pressure	10005750 Increase in blood pressure	10022891 Laboratory and instrumental data	N	Recovery without any consequences	Possible	N
28	R075	G5	Nausea, vomiting	10047700 Vomit	10017947 Gastrointestinal disorders	N	Recovery without any consequences	Possible	N
29	R075	G5	COVID-19	10084268 Coronavirus infection COVID-19	10021881 Infections and infestations	N	Recovery without any consequences	Doubtful	Y
30	R077	G5	Headache	10019211 Headache	10029205 Nervous system disorders	N	Recovery without any consequences	Possible	N
31	R077	G5	Acute intestinal infections (AII). Acute gastroenteritis of moderate severity. Dehydration 0-1 tbsp.	10017888 Gastroenteritis	10021881 Infections and infestations	N	Recovery without any consequences	Doubtful	Y
32	R078	G3	COVID-19	10084268 Coronavirus infection COVID-19	10021881 Infections and infestations	N	Recovery without any consequences	Doubtful	Y
33	R080	G3	Increase in the number of leukocytes. An increase in the absolute and relative number of eosinophils	10014945 Increase in the number of eosinophils	10022891 Laboratory and instrumental data	N	Recovery without any consequences	Doubtful	N
34	R080	G3	Acute enterocolitis. Diarrhea	10014893 Enterocolitis	10017947 Gastrointestinal disorders	N	Recovery without any consequences	Doubtful	Y
35	R090	G5	Lymphocytosis	10025280 Lymphocytosis	10005329 Blood and lymphatic	N	Recovery without any consequences	Possible	N

					system disorders				
36	R090	G5	Increase in the alanine aminotransferase levels (ALT)	10001551 Increase in the alanine aminotransferase levels (ALT)	10022891 Laboratory and instrumental data	N	Recovery without any consequences	Possible	N
37	R097	G3	Nasal congestion	10028735 Nasal congestion	10038738 Respiratory, thoracic and mediastinal disorders	N	Recovery without any consequences	Possible	N
38	R097	G3	Leukocytosis	10024378 Leukocytosis	10005329 Blood and lymphatic system disorders	N	Recovery without any consequences	Possible	N
39	R098	G5	Lymphocytosis	10025280 Lymphocytosis	10005329 Blood and lymphatic system disorders	N	Recovery without any consequences	Possible	N
40	R098	G5	Neutropenia	10029354 Neutropenia	10005329 Blood and lymphatic system disorders	N	Recovery without any consequences	Possible	N
41	R098	G5	Increase in the alanine aminotransferase levels (ALT)	10001551 Increase in the alanine aminotransferase levels (ALT)	10022891 Laboratory and instrumental data	N	Recovery without any consequences	Possible	N
42	R098	G5	COVID-19	10084268 Coronavirus infection COVID-19	10021881 Infections and infestations	N	Recovery without any consequences	Doubtful	Y
43	R104	G3	Lymphocytosis	10025280 Lymphocytosis	10005329 Blood and lymphatic system disorders	N	Recovery without any consequences	Possible	N
44	R104	G3	Neutropenia	10029354 Neutropenia	10005329 Blood and lymphatic system disorders	N	Recovery without any consequences	Possible	N
45	R104	G3	Increase in the level of total bilirubin	10005364 Increase in the bilirubin levels	10022891 Laboratory and instrumental data	N	Recovery without any consequences	Possible	N
46	R109	G3	Lymphocytosis	10025280 Lymphocytosis	10005329 Blood and lymphatic system disorders	N	Recovery without any consequences	Possible	N
47	R109	G3	Acute respiratory viral infection	10047461 Viral infection	10021881 Infections and infestations	N	Recovery without any consequences	Possible	N
48	R114	G5	Acute respiratory viral infection / Nasal congestion, (tickling) sore throat, Weakness	10047461 Viral infection	10021881 Infections and infestations	N	Recovery without any consequences	Possible	N

49	R115	G3	Decrease in the relative number of segmented neutrophils	10029366 Decrease in the number of neutrophils	10022891 Laboratory and instrumental data	N	Unknown	Possible	N
